# The impact of COVID-19 on the injury pattern for maxillofacial fracture in Daegu city, South Korea

**DOI:** 10.1186/s40902-021-00322-6

**Published:** 2021-09-13

**Authors:** Dong-Woo Lee, So-Young Choi, Jin-wook Kim, Tae-Geon Kwon, Sung-Tak Lee

**Affiliations:** Department of Oral and Maxillofacial Surgery, School of Dentistry, Kyungpook National University, 2177 Dalgubeol-daero, Jung-gu, Daegu, 41940 Republic of Korea

**Keywords:** COVID-19, Corona, Pandemic, Oral and maxillofacial fracture, Facial fracture

## Abstract

**Background:**

This study aimed to analyze the impact of COVID-19 on oral and maxillofacial fracture in Daegu by comparing the demographic data in 2019 and 2020, retrospectively. We collected data from all patients having trauma who visited the emergency room for oral and maxillofacial fractures.

**Methods:**

This retrospective study was based on chart review of patients who visited the emergency department of Kyungpook National University Hospital in Daegu, South Korea from January 1, 2019, to December 31, 2020. We conducted a comparative study for patients who presented with maxillofacial fractures with occlusal instability during pre-COVID-19 era (2019) and COVID-19 era (2000) with demographics and pattern of injuries.

**Results:**

After the outbreak of COVID-19, the number of monthly oral and maxillofacial fractures, especially sports-related oral and maxillofacial fractures, decreased significantly. Also, the number of alcohol-related fractures increased significantly. In addition, as the number of monthly confirmed cases of COVID-19 increases, the incidence of fracture among these cases tends to decrease.

**Conclusions:**

The COVID-19 pandemic has changed the daily life in Korea. Identifying the characteristics of patients having trauma can provide a good lead to understand this long-lasting infectious disease and prepare for future outbreaks.

## Background

Coronavirus disease 2019 (COVID-19), which is caused by severe acute respiratory syndrome coronavirus 2 (SARS-CoV-2), is an infectious disease that first originated in Wuhan, China, in December 2019 [1]. This disease is highly contagious, such that it was declared a pandemic on March 11, 2020, by the World Health Organization due to its rapid spread worldwide [2].

COVID-19 has caused an astronomical number of infections and deaths, causing the collapse of health care systems worldwide. To overcome this situation, social restrictions have been placed by governments around the world. Institutional restrictions are placed on the basis of wearing masks and social distancing [3, 4]. For the care of COVID-19-infected patients in the medical field, compulsory changes have been effected in elective surgery and out-patients clinics. Therefore, COVID-19 led to a significant reduction in the number of patients hospitalized, as compared to the parallel periods of previous years [5, 6].

Nevertheless, trauma remains a major health care concern even during periods of high-impact disease outbreaks, since it cannot be easily predicted, considering that it is influenced by various personal and social factors and that it occurs incidentally. Injury patterns are known to be closely related to human behavior (both injury preventive and inducing behavior). A major change in behavioral patterns of the public due to the direct impact of COVID-19 led to changes in the regularly observed injury patterns [7–9].

Similarly, the etiology of maxillofacial traumas changed during the first COVID-19 lockdown period. Maxillofacial trauma varies from region to region, but usually occurs due to fall-downs, assaults, car accidents, vehicle collisions, and sports-related injuries. The causes of trauma have changed and various countries have reported these changes [10–13].

As far as we know, there is no report on traumatic changes in the maxillofacial region in South Korea. Therefore, this study aimed to present the etiology of trauma in the maxillofacial region in Daegu, South Korea.

Daegu is the third largest city in Korea and the place where the first large outbreak of COVID-19 occurred in South Korea. After the first confirmed case of COVID-19 was announced in January 20, 2020, there was a large steep spike of the number of cases in Daegu in February 18, 2020. Since then, the number of COVID-19 patients in South Korea has increased rapidly [14, 15]. In addition, since our hospital is the only hospital in Daegu responsible for maxillofacial trauma, it is judged as suitable for investigating the etiology of maxillofacial trauma changes in the early stage of COVID-19 outbreak.

## Methods

This retrospective study was based on chart review of patients who visited the emergency department of Kyungpook National University Hospital in Daegu, South Korea, from January 1, 2019, to December 31, 2020. Approval for this study was granted by the Institutional Review Board of Kyungpook National University Dental Hospital (KNUDH-2021-07-04-00).

The inclusion criteria for this study were as follows: (1) all patients presenting during this period for evaluation and treatment to the Department of Oral and Maxillofacial Surgery and (2) patients with maxillofacial fractures who complained of occlusal instability or clearly observed occlusal instability at the doctor’s decision. Patients with uncertain cause of fracture were excluded.

We conducted a comparative study by dividing the patients into two groups: patients during the COVID-19 period (year 2020, experimental group) and before the COVID-19 period (year 2019, control group).

In this study, the following demographic data were collected: patient’s age, gender, date of injury, injury etiology, and alcohol relation.

The injury etiology was subdivided into seven main categories: assault, fall-downs, motor-vehicle accident (MVA), sports, labor, high fall, and accidents. The assault category included traumas resulting from human violence. The fall-downs category was considered traumas resulting from self-injury via stumble. The MVA included traffic accidents involving cars, motorcycles, bicycles, electric scooter (E-scooter), and electric wheelchair (E-wheelchair). The sports category was considered traumas suffered during physical activities and the labor category included traumas suffered during work. The high fall category was considered a fall from a high top (over 3 m) to the ground. The accidents’ category is unintentional accidents not belonging to the following six categories (e.g., fall of heavy object on patient).

The number of fracture patients per month and age group was analyzed by independent T-test. The median age between 2 years was compared by Wilcoxon rank-sum test. The proportional change was compared by two-proportion z-test. The confirmed cases and fracture patients were evaluated by Spearman correlation analysis. Statistical significance was determined as *p* < 0.05 and a 95% confidence interval was considered valid. All statistical analysis was performed using SPSS version 25.0 (IBM Corp., Armonk, NY, USA).

## Results

### Demographics of oral and maxillofacial fracture patients

The total number of patients who received treatment at the emergency department of Kyungpook National University Hospital for oral and maxillofacial fractures due to maxillofacial trauma was 253 and 194 in 2019 and 2020, respectively. The total number of patients during the COVID-19 period decreased by 23.3% and the monthly average number of fracture patients in 2020 significantly decreased when compared to 2019 (*p* = 0.030, Table 1).

**Table 1:** Demographics of oral and maxillofacial fracture patients during 2020 versus 2019

	2019	2020	*P*
Total	253	194	
Average per month	21.08± 4.98	16.17± 7.02	0.030*
Gender	M 186 (73%)	M 147 (76%)	0.294
	F 67 (26%)	F 47 (24%)	
Alcohol related	58 (23%)	60 (31%)	0.029*
Age (median)	38 (23–56)	34 (22–53.25)	0.162
Age 0~19	3.42 ± 2.19	2.67 ± 1.44	0.166
Age 20~39	7.50 ± 2.47	6.50 ± 2.97	0.190
Age 40~59	5.92 ± 3.12	4.60 ± 2.22	0.139
Age 60 +	4.25 ± 2.26	3.80 ± 1.81	0.309

*Significant at 95% confidence level

The gender ratio of fracture patients in 2019 was 73.52% male and 26.48% female in 2019; and 75.77% male and 24.23% female in 2020, indicating that the change in the trauma ratio according to gender was not significant (*p* = 0.294). The median age of patients was 38 years in 2019 and 34 years in 2020, indicating no significant difference (*p* = 0.162). When dividing the groups into age ranges, including 0–19, 20–39, 40–59, and 60 or older, there were no significant differences observed based on all the age groups (*p* > 0.05).

**Fig. 1:**
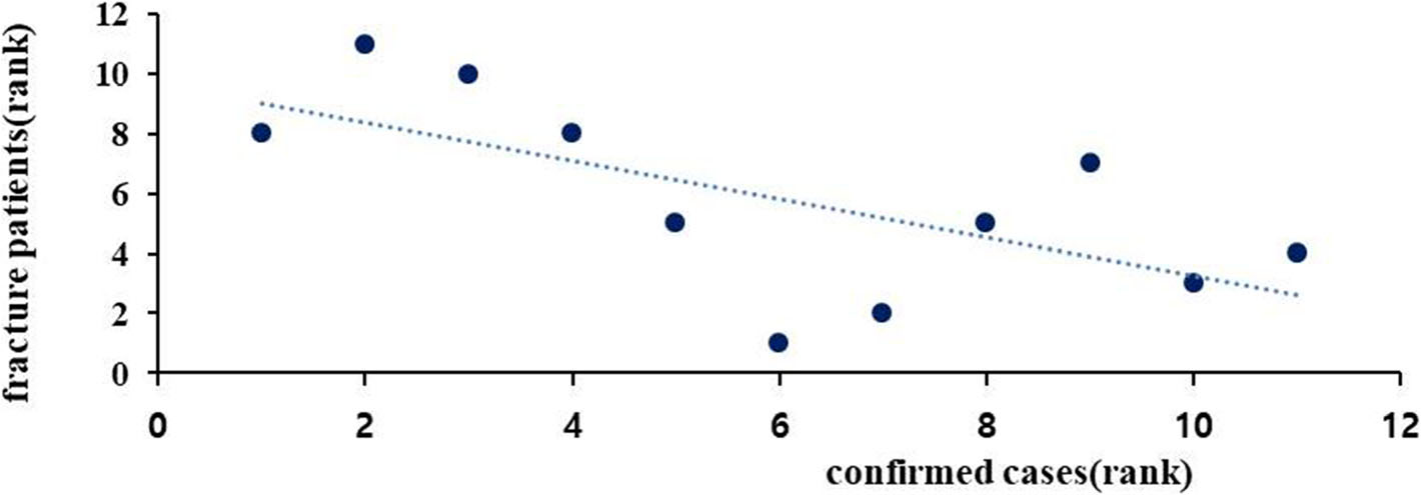
Correlation between COVID-19 confirmed cases and oral and maxillofacial fracture patients during 2020 (*r* = − 0.667, *P* = 0.025)

**Fig. 2:**
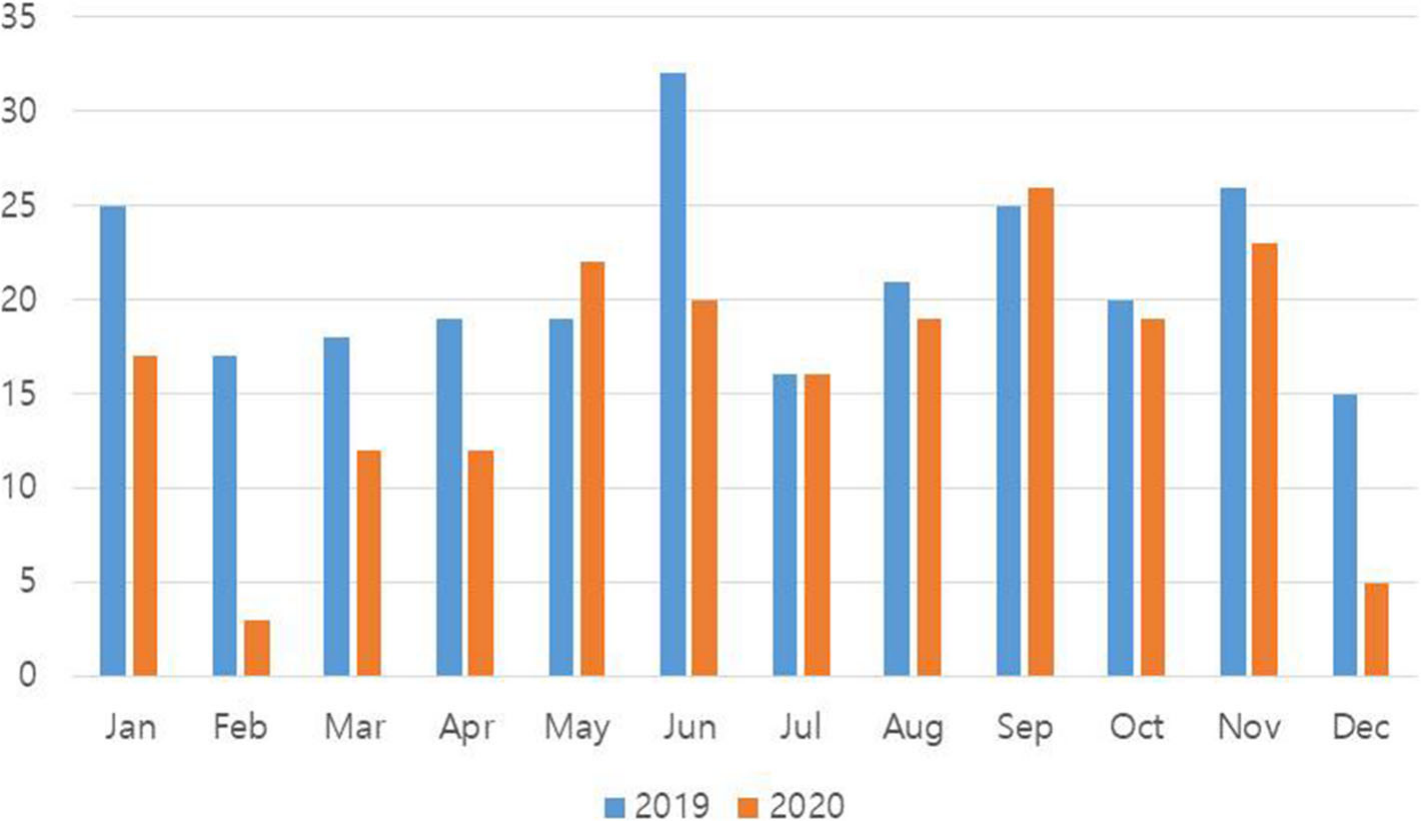
Monthly number of oral and maxillofacial fracture patients during 2019 and 2020

There was a significant increase in the proportion of alcohol-fractures in 2020 (30.93%) compared to 2019 (22.92%) (*p* = 0.029). The correlation between the number of COVID-19 confirmed cases and the number of fracture patients was investigated from February (when the first confirmed case was detected in Daegu) to December. There was a significantly negative correlation between the two variables (*r* = −0.667; *p* = 0.025), which indicates that as the monthly number of confirmed cases decreased, the monthly number of fracture patients tends to increase (Fig. 1). When comparing the monthly number of fracture patients, the number of fracture patients in February, June, and December was remarkable (Fig. 2).

### Etiology of oral and maxillofacial fracture patients

During 2020, a decrease in the number of patients in all categories, except the labor category, was seen. There was no significant difference in the proportion of fall-downs, MVA, assault, accidents, labor, and high fall (*p* > 0.05). In contrast, there was a significant decrease in the proportion of sports category (*p* = 0.019, Table 2).

**Table 2:** Etiology and type of oral and maxillofacial fracture patients during 2020 versus 2019

	2019	2020	***P***
**Etiology (%)**			
Accident	13 (5%)	8 (4%)	0.308
High fall	4 (2%)	1 (1%)	0.144
Laboral	10 (4%)	13 (7%)	0.096
Fall down	107 (42%)	88 (45%)	0.258
Sports	11 (4%)	2 (1%)	0.019*
Assault	40 (16%)	32 (16%)	0.423
MVA (% in total MVA)	68 (27%)	50 (26%)	0.396
Motorcycle	*15 (22%)*	*16 (32%)*	*0.025*
Car	*28 (41%)*	*14 (28%)*	*0.140*
Bicycle	*17 (25%)*	*14 (28%)*	*0.714*
E-scooter	*7 (10%)*	*6 (12%)*	*0.770*
E-wheelchair	*1 (2%)*	*0 (0%)*	*0.389*
**Type (%)**			
Mn.sym.	80 (41%)	96 (38%)	0.66
Mn.body	25 (13%)	40 (16%)	0.55
Mn.angle	51 (26%)	70 (28%)	0.75
Mn.ramus	7 (4%)	13 (5%)	0.73
Intracapsular	54 (28%)	85 (34%)	0.36
Extracapsular	50 (26%)	42 (17%)	0.12
Coronoid	4 (2%)	4 (2%)	1.00
Lefort1 fx.	6 (3%)	11 (4%)	0.70
Lefort2 fx.	7 (4%)	8 (3%)	0.70
ZMC	9 (5%)	8 (3%)	0.47

*Significant at 95% confidence level

When the MVA was classified according to means of transportation, the decrease in the proportion of MVA by car was remarkable in 2020 compared to 2019 (41.18% versus 28%); however, there were no significant differences between these time periods in all other means of transportation.

### Correlation between means of transportation and oral and maxillofacial fractures

Data from 2019 and 2020 were combined to investigate the oral and maxillofacial fracture pattern according to the means of transportation (Table 3). In motorcycle accidents, mandible symphysis was the most common fracture site. The next most common site was mandible body and intracapsular condyle. Mandible symphysis fracture was the most common in car accident, while mandible angle and body was the second most common fracture. In bicycle and E-scooter, intracapsular condyle fracture was the most common fracture and mandible symphysis was the second. There was a significant relationship with fracture by E-scooter and intracapsular condyle fracture (*p* = 0.013).

**Table 3:** MVA (motor vehicle accidents) and oral and maxillofacial fracture site

	Motorcycle	Car	Bicycle	E-scooter
Mn.sym.	13 (42%)	19 (45%)	11 (35.48%)	6 (46%)
Mn.angle	4 (13%)	11 (26%)	2 (6%)	0 (0%)
Intracapsular condyle	8 (26%)	7 (177%)	14 (45%)	8 (62%)*
Extracapsular condyle	5 (16%)	9 (21%)	8 (26%)	3 (23.%)
Mn.ramus	3 (10%)	2 (5%)	0 (0%)	0 (0%)
Coronoid	0 (0%)	1 (2%)	1 (3%)	0 (0%)
Mn.body	9 (29%)	11 (26%)	4 (13%)	0 (0%)
Lefort 1	2 (6%)	4 (10%)	2 (6.45%)	1 (8%)
Lefort2	1 (3%)	6 (14%)	1 (3.23%)	0 (0%)
ZMC	1 (3.23%)	4 (9.5210%)	1 (3.23%)	1 (8%)

*Significant at 95% confidence level

## Discussion

The causes of trauma are closely related to human behavior. Institutional restrictions, such as social distancing due to the precedented COVID-19 pandemic, have had a great impact on the changes in the behavior of the public and this has also changed the pattern of trauma.

The number of patients with trauma-induced oral and maxillofacial fractures has also been affected and etiology changes were also observed. There were no significant differences in gender, median age, and number of patients according to age group. However, the monthly number of fracture patients decreased significantly, and in particular, the number of fractures due to sports significantly decreased. Suggestively, these decreases are thought to be due to the decrease in outdoor activities, such as sports due to social distancing in the COVID-19 pandemic [16, 17].

Conversely, although no statistically significant difference was seen, the proportion of assault was found to be higher than before the COVID-19 pandemic period. This indicates that interpersonal violence increased with the outbreak of COVID-19. Some studies have also shown that domestic violence and violence against women and children have increased after the COVID-19 outbreak [18, 19].

In addition, there has been a significant increase in the number of oral and maxillofacial fracture patients per month related to alcohol-drinking during 2020 compared to 2019. Some studies revealed that interpersonal violence and intimate partner violence increased with changes in alcohol consumption habits [20, 21]. Due to the restrictive measures, such as ban on business after 9 p.m. and ban on gatherings of more than five people, harmful alcohol consumption at home increased [22, 23]. The pattern of trauma, especially those related with assault, also changed according to the change in alcohol consumption habits during COVID-19. Therefore, when treating maxillofacial trauma patients, especially women and children, in relation to the increase in violence and alcohol consumption, doctors should pay additional attention to ensure their safety at home.

There was no significant decrease in 2020 compared with 2019 in MVA; however, a decreased incidence of car accidents was observed. This is assumed to be a phenomenon caused by a decrease in long distance movement and travel [24.]

Since there is a significantly negative correlation between the number of confirmed cases and the number of fracture patients, as the monthly number of confirmed cases increases, the number of fracture patients tends to decrease. It is interpreted that, as the number of confirmed cases increases, government policies, such as strengthening of social distancing and reduction of outdoor activities and citizens’ movement, become more strict. When comparing the monthly number of fracture patients, the months with the largest difference were February, June, and December. February 2020 was the month when the first confirmed case in Daegu was reported, whereas June and December 2020 were the months when the level of social distancing was raised due to a surge in the number of confirmed cases [3, 24]. Judging from this, it seems that the number of patients with fractures decrease according to the government’s policy as the number of confirmed cases increase.

Fracture by personal means of transportation, such as motorcycles, bicycles, and electric kickboards did not decrease in spite of the COVID-19 pandemic. This may mean that there is a tendency of using transportation with less human contact rather than public transportation involving many people-to-people contact. In particular, there is a statistically significant correlation between maxillofacial fractures induced by electric kickboards and intracapsular condyle fractures. Although the electric kickboard is a convenient means of transportation, it must be used with caution when considering complications that affect the quality of life, such as malocclusion and mouth open restriction due to temporomandibular joint ankylosis after intracapsular condyle fracture.

This study has several limitations. First, it was limited to patients with maxillofacial trauma with occlusal instability. In the future studies, patients with trauma that are maintaining occlusal stability, such as nasal bone fracture and periorbital fracture, should be included. Second, this study is limited to the early stage of COVID-19, that is, before the social changes caused by government restriction were completely settled. In future studies, data from 2021 when social changes due to COVID-19 are settled should be included.

## Conclusion

After the outbreak of COVID-19, as the number of monthly COVID-19 confirmed cases increases, the number of monthly fracture patients tends to decrease. Also, the number of monthly oral and maxillofacial fractures and sports-related oral and maxillofacial fractures significantly decreased and the number of alcohol-related fractures significantly increased.

The COVID-19 pandemic has been around the world since early 2020 and countries are making various efforts to prevent the continuous spread of the infection. In South Korea, efforts were made to prevent the spread of infection, such as bans on gatherings of more than five people, bans on business after 9 p.m., non-face-to-face classes, and encouragement to frequently wear masks and wash hands. Even under these circumstances, the pandemic has lasted for a long time. Therefore, identifying the characteristics of maxillofacial trauma patients can be a good lead to prevent the fracture incidence and appropriately manage the patients with maxillofacial trauma in this COVID-19 era.

## Data Availability

Not applicable. (Data sharing is not applicable to this article as no datasets were generated or analyzed during the current study.)

## Abbreviations

COVID-19: Coronavirus disease 2019

## References

[1] et al (2020) An mRNA vaccine against SARS-CoV-2 - preliminary report. N Engl J Med 383(20):1920–1931. 10.1056/NEJMoa2022483Jackson LA, Anderson EJ, Rouphael NGet al (2020) An mRNA vaccine against SARS-CoV-2 - preliminary report. N Engl J Med 383(20):1920-1931. doi:10.1056/NEJMoa2022483 10.1056/NEJMoa2022483 PMC7377258 32663912

[2] Jee Y (2020). WHO international health regulations emergency committee for the COVID-19 outbreak. Epidemiol Health.

[3] Ahmed F, Zviedrite N, Uzicanin A (2018). Effectiveness of workplace social distancing measures in reducing influenza transmission: a systematic review. BMC Public Health.

[4] Nonpharmaceutical interventions (2021) Centers for Disease Control and Prevention, centers for disease control and Prevention. Available at: http://www.cdc. gov/nonpharmaceutical-interventions/index.html. Accessed 15 July, 2021.

[5] Allevi F, Dionisio A, Baciliero U, Balercia P, Beltramini GA, Bertossi D, Bozzetti A, Califano L, Cascone P, Colombo L, Copelli C, de Ponte FS, de Riu G, Della Monaca M, Fusetti S, Galié M, Giannì AB, Longo F, Mannucci N, Nocini PF, Pelo S, Ramieri G, Sesenna E, Solazzo L, Spinelli G, Tarsitano A, Tartaro G, Valentini V, Verrina G, Biglioli F (2020). Impact of COVID-19 epidemic on maxillofacial surgery in Italy. Br J Oral Maxillofac Surg.

[6] Horan J, Duddy JC, Gilmartin B, Amoo M, Nolan D, Corr P, Husien MB, Bolger C (2021) The impact of COVID-19 on trauma referrals to a National Neurosurgical Centre. Ir J Med Sci. 10.1007/s11845-021-02504-7 10.1007/s11845-021-02504-7 PMC7790516 33415689

[7] de Boutray M, Kün-Darbois JD, Sigaux N, Lutz JC, Veyssiere A, Sesque A, Savoldelli C, Dakpe S, Bertin H, Lallemant B, Llobet A, du Cailar M, Lauwers F, Davrou J, Foletti JM (2021). Impact of the COVID-19 lockdown on the epidemiology of maxillofacial trauma activity: a French multicentre comparative study. Int J Oral Maxillofac Surg.

[8] Lalloo R, Lucchesi LR, Bisignano C, Castle CD, Dingels ZV, Fox JT, Hamilton EB, Liu Z, Roberts NLS, Sylte DO, Alahdab F, Alipour V, Alsharif U, Arabloo J, Bagherzadeh M, Banach M, Bijani A, Crowe CS, Daryani A, Do HP, Doan LP, Fischer F, Gebremeskel GG, Haagsma JA, Haj-Mirzaian A, Haj-Mirzaian A, Hamidi S, Hoang CL, Irvani SSN, Kasaeian A, Khader YS, Khalilov R, Khoja AT, Kiadaliri AA, Majdan M, Manaf N, Manafi A, Massenburg BB, Mohammadian-Hafshejani A, Morrison SD, Nguyen TH, Nguyen SH, Nguyen CT, Olagunju TO, Otstavnov N, Polinder S, Rabiee N, Rabiee M, Ramezanzadeh K, Ranganathan K, Rezapour A, Safari S, Samy AM, Sanchez Riera L, Shaikh MA, Tran BX, Vahedi P, Vahedian-Azimi A, Zhang ZJ, Pigott DM, Hay SI, Mokdad AH, James SL (2020). Epidemiology of facial fractures: incidence, prevalence and years lived with disability estimates from the global burden of disease 2017 study. Inj Prev.

[9] Salzano G, Dell'Aversana Orabona G, Audino G (2020). Have there been any changes in the epidemiology and etiology of maxillofacial trauma during the COVID-19 pandemic? An Italian multicenter study. J Craniofac Surg.

[10] Dillon JK, Christensen B, McDonald T, Huang S, Gauger P, Gomez P (2021). The financial burden of mandibular trauma. J Oral Maxillofac Surg.

[11] Boffano P, Kommers SC, Karagozoglu KH, Forouzanfar T (2014). Aetiology of maxillofacial fractures: a review of published studies during the last 30 years. Br J Oral Maxillofac Surg.

[12] Rozenfeld M, Peleg K, Givon A, Bala M, Shaked G, Bahouth H, Bodas M (2021) COVID-19 changed the injury patterns of hospitalized patients. Prehosp Disaster Med. 36(3):251-259. doi: 10.1017/S1049023X21000285. Epub 2021 Mar 1. PMID: 33641689; PMCID: PMC7985901. 10.1017/S1049023X21000285 PMC7985901 33641689

[13] Wang CJ, Hoffman GR, Walton GM (2021). The implementation of COVID-19 social distancing measures changed the frequency and the characteristics of facial injury: the Newcastle (Australia) experience. Craniomaxillofac Trauma Reconstr.

[14] Her M (2020). How is COVID-19 affecting South Korea? What is our current strategy?. Disaster Med Public Health Prep.

[15] Kim JH, An AR, Min PK, Bitton A, Gawande AA (2020). How South Korea responded to the COVID-19 outbreak in Daegu. NEJM Catal Innov Care Deliv.

[16] Yeo TJ (2020). Sport and exercise during and beyond the COVID-19 pandemic. Eur J Prev Cardiol.

[17] Evans DP (2020). COVID-19 and violence: a research call to action. BMC Womens Health.

[18] Sri AS, Das P, Gnanapragasam S, Persaud A (2021) COVID-19 and the violence against women and girls: 'The shadow pandemic'. Int J Soc Psychiatry. 10.1177/0020764021995556 10.1177/0020764021995556 33593144

[19] Pollard MS, Tucker JS, Green HD (2020). Changes in adult alcohol use and consequences during the COVID-19 pandemic in the US. JAMA Netw Open.

[20] Kim K (2021). Impacts of COVID-19 on transportation: summary and synthesis of interdisciplinary research. Transp Res Interdiscip Perspect.

[21] Krishnakumar A, Verma S (2021) Understanding domestic violence in India during COVID-19: a routine activity approach [published online ahead of print, 2021 Mar 10]. Asian J Criminol. 1-17. doi:10.1007/s11417-020-09340-1 10.1007/s11417-020-09340-1 PMC7945968 33723492

[22] Grossman ER, Benjamin-Neelon SE, Sonnenschein S (2020). Alcohol consumption during the COVID-19 pandemic: a cross-sectional survey of US adults. Int J Environ Res Public Health.

[23] Barbara G, Facchin F, Micci L, Rendiniello M, Giulini P, Cattaneo C, Vercellini P, Kustermann A (2020). COVID-19, lockdown, and intimate partner violence: some data from an Italian service and suggestions for future approaches. J Women's Health (Larchmt).

[24] Pandey A, Saxena NK (2020). Effectiveness of government policies in controlling COVID-19 in India. Int J Health Serv.

